# The Intersections of COVID-19 Global Health Governance and Population Health Priorities: Equity-Related Lessons Learned From Canada and Selected G20 Countries

**DOI:** 10.3389/phrs.2024.1606052

**Published:** 2024-01-29

**Authors:** Muriel Mac-Seing, Erica Di Ruggiero

**Affiliations:** ^1^ Department of Social and Preventive Medicine, School of Public Health & Centre de Recherche en Santé Publique, Université de Montréal, Montreal, QC, Canada; ^2^ Behavioural Health Sciences Division, Centre for Global Health, Dalla Lana School of Public Health, University of Toronto, Toronto, ON, Canada; ^3^ Centre for Global Health, Dalla Lana School of Public Health, University of Toronto, Toronto, ON, Canada; ^4^ Division of Social and Behavioural Health Sciences, Dalla Lana School of Public Health, University of Toronto, Toronto, ON, Canada; ^5^ Institute of Health Policy Management and Evaluation, Dalla Lana School of Public Health, University of Toronto, Toronto, ON, Canada

**Keywords:** COVID-19, equity, global health governance, population health priorities, Canada and G20

## Abstract

**Background:** COVID-19-related global health governance (GHG) processes and public health measures taken influenced population health priorities worldwide. We investigated the intersection between COVID-19-related GHG and how it redefined population health priorities in Canada and other G20 countries. We analysed a Canada-related multilevel qualitative study and a scoping review of selected G20 countries. Findings show the importance of linking equity considerations to funding and accountability when responding to COVID-19. Nationalism and limited coordination among governance actors contributed to fragmented COVID-19 public health responses. COVID-19-related consequences were not systematically negative, but when they were, they affected more population groups living and working in conditions of vulnerability and marginalisation.

**Policy options and recommendations:** Six policy options are proposed addressing upstream determinants of health, such as providing sufficient funding for equitable and accountable global and public health outcomes and implementing gender-focused policies to reduce COVID-19 response-related inequities and negative consequences downstream. Specific programmatic (e.g., assessing the needs of the community early) and research recommendations are also suggested to redress identified gaps.

**Conclusion:** Despite the consequences of the COVID-19 pandemic, programmatic and research opportunities along with concrete policy options must be mobilised and implemented without further delay. We collectively share the duty to act upon global health justice.

## Background

The COVID-19 pandemic offered three main lessons: 1) although the warning signs of an emerging international zoonotic health crisis were there, the global community and national governments were not prepared to effectively respond to COVID-19; 2) amid rapid policy and decision-making processes, both global health governance (GHG) and national governments failed to promote global health solidarity and ensure equity in health outcomes among their constituencies; and 3) population groups working and living in conditions of vulnerability and marginalisation experienced further gender, racial, socioeconomic, and health inequities exacerbated by COVID-19 as a result of governance processes and public health measures adopted [1]. Global health governance refers to “governance arrangements needed to further agreed global health goals” such as health equity and access to essential medicines including vaccines among GHG actors such as the World Health Organization (WHO), national governments, and civil society organisations [2]. Given the virus’ rapid spread worldwide and the high number of COVID-19-related deaths reported in Europe and the USA in the first year of the pandemic, biomedical efforts were mainly deployed to control its transmission and manage the severity of the disease [3], while equity considerations were not sufficiently prioritised [4]. In 2021, the second year of the pandemic, global pharmaceutical efforts were mobilised at an unprecedented speed toward the COVID-19 vaccine development, approval, and rollout [5] and the launching of the COVID-19 Vaccines Global Access (COVAX) initiative, spearheaded by WHO to foster global vaccine equity [6].

In parallel, research activities increased exponentially in a short period to address the numerous emerging challenges created by COVID-19. A recent study mapped more than 17,900 COVID-19 research projects from 157 countries, with more than half of the projects conducted in the UK (4,460), the USA (3,953), and Canada (1,772) [7]. Projects examined different topics that included SARS-CoV-2, its transmission, diagnostics and clinical management, candidate vaccines, COVID-19 public health measures taken and their consequences, and communication issues [7]. This sudden focus on COVID-19 led to the “covidization” of research priorities, a process where financial, human, and technical resources are channelled to address COVID-19 [8]. Although it is essential to swiftly respond to the COVID-19 pandemic including through research, little is known about how and to what extent the COVID-19-related GHG is affecting population health including research in high-resource countries such as Canada. We hence examined the intersection between COVID-19-related GHG and population health research priorities in Canada and selected G20 countries. Specifically, we aimed to describe the features of COVID-19-related GHG in Canada and their impacts on influencing research agendas (such as objectives, scope, and collaboration) and explored the consequences on research projects’ population health aspects (such as social determinants of health and equity). We also investigated these research objectives at level of the G20 countries to contrast what was found in Canada.

### Methods

To address these research objectives, we conducted a multi-method analysis of COVID-19-related GHG features on population health research in Canada. First, we conducted a multilevel qualitative study from the perspective of four groups of actors (researchers, research funders, and global/public health centres/institutes from Canada, and WHO/international actors) [9]. Our qualitative data was informed by the Intersectionality-Based Policy Analysis (IBPA) framework, which makes visible less visible different types of inequities (e.g., gender, racial, socioeconomic, disability, region, and others) [10], and the Multiple Streams framework (MSF), which analyses the problem, policy, and political streams and windows of opportunity for policy change [11]. Two sets of research questions were asked, descriptive questions related to both the IBPA [10] and MSF questions to identify the problem and windows of opportunity [11] when examining the relationships between COVID-19-related GHG and population health research priorities in Canada, and transformative questions related to solutions and recommendations proposed by study respondents to address identified problems [10]. Qualitative data were collected from February to August 2022 through in-depth semi-structured interviews of 60 min, conducted in English and French (MMS). Given our commitment to include respondents with a diversity of social identities and experiences, we conducted purposive sampling to maximise variation while considering gender, ethnicity, discipline, career seniority, and geography [12]. Recruitment of participants continued until thematic saturation was reached [12]. In total, 35 respondents were interviewed: 18 researchers from seven Canadian provinces, four Canadian global/public health and health policy research centres/institutes, nine Canadian research funding agency representatives, and four WHO/international actors. Seventy-five percent (25/35) of respondents were women and 20% (7/35) identified as Black, Indigenous/Métis, or People of Color. During the 2022 Canadian Conference on Global Health, we also conducted a 90-minute interactive workshop involving 40 people who participated in person and virtually to identify further recommendations.

Second, for the scoping review [13], we identified peer-reviewed literature that focused on five concepts: COVID-19, GHG, population health, equity, and the G20 countries. We followed Arksey’s & O’Malley’s five-stage scoping review framework [14] and PRISMA Extension for Scoping Reviews (PRISMA-ScR) Checklist [15]. We used the Population, Concept, and Context (PCC) framework for scoping reviews [16]. Supplementary Annex S1 details the search terms and keywords we used. We searched peer-reviewed journals in Medline, Global Health, Web of Science, and Embase in English and French, from January 2020 to April 2023. Inclusion criteria included original research, reviews, and commentaries that addressed implicitly or explicitly GHG, equity, and population health priorities (policy, programme, and research) in any of the G20 countries. Out of 6,254 references identified, 14 studies met the inclusion criteria and were included in the last phase (Supplementary Annex S2). Fourteen G20 countries and regions included in the reviewed references were Australia, Brazil, Canada, China, France, Italy, India, Japan, Russia, South Africa, the UK, the USA, and the European Union (EU) and BRICS (Brazil, Russia, India, China, and South Africa) regions.

## Evidence

We report the cross-examination of evidence related to the Canadian and G20 components of our research, followed by policy options and recommendations for programmes and research.

### Features of COVID-19-Related GHG, Challenges and Opportunities

One of the main features of COVID-19-related GHG described by the Canadian-based qualitative study respondents was its ‘messiness’. This is due to the multitude of actors involved in addressing COVID-19 in addition to existing global health issues, but also because of limited coordination and accountability among key GHG actors.

I’ll start by saying it’s a mess. And that’s partly because we are in a global health crisis. But the other two reasons it’s messy is because first of all there are just a lot of different actors, not only all of the different members of the World Health Organization, so all the countries, but then we have intergovernmental organisations like the World Trade Organization, GAVI, CEPI, the list goes on, and so we have to figure out what are their relationships with one another, who makes decisions (…). The other thing that makes it really messy is there’s no sort of established framework of accountability for how people should respond or make decisions in the pandemic. (Canadian Researcher Respondent 5)

In addition to addressing the numerous emerging challenges created by COVID-19, many governments including Canada were caught between promoting globalism in a time of a planetary health crisis and prioritising the health of their citizens and national economies.

We have seen global views and views of solidarity between countries but at the same time we saw very state-centric approaches with vaccine nationalism for those very same countries, especially developed countries, OECD countries were closing their borders, hoarding vaccines, exercising pre-purchase agreements on vaccines (…). (WHO Respondent 32)

At the G20 level, inward-looking behaviours were illustrated through insufficient collaboration and coordination among actors at global, regional, national, and sub-national levels of governance [13]. These governance actors did not align their priorities when implementing different COVID-19 public health measures (e.g., border closure, COVID-19 testing and tracing, and quarantine measures), generating further fragmentation among countries sharing common borders such as in Northern Ireland (the UK) and the Republic of Ireland [17], or within the same country such as in Brazil [18]. Although local authorities and civil society organisations (CSO) demonstrated a lack of coordination in their COVID-19 response activities, CSOs in the EU used creative approaches to reach their constituencies such as providing online services and SIM cards to youth in situations of vulnerability during lockdowns to deliver essential social services [19].

Despite these challenges, specific opportunities were also reported that addressed global solidarity through further multilateral action for promoting equity.

There’s a real opportunity for Canada to translate its support of multilateralism into concrete action and that is through the WHO relationship. WHO is at the heart of the multilateral system. And so if Canada were, for example, to learn lessons from Germany, but to be a stronger multilateralist in word and deed in the important fora in which Canada participates, the G7, the G20, the Commonwealth, the Francophonie, and I think the partnership with WHO which is at the centre of the multilateral system in health is a good way to commit with that. And by the way, some of those fundamental issues going from equity to solidarity to empathy are not unique to the pandemic. (WHO Canadian Respondent 13)

### Equity at the Intersection of COVID-19-Related GHG and Population Health Research Priorities

Despite the imperatives to control COVID-19 and save lives, respondents signaled that global governance including in global health collectively failed to address equity, instead penalised actors that conducted initiatives that promoted global equity.

The global governance system for research and equity failed. The surveillance system was better than probably one expects though it is unbelievably punishing, as you know, to have [RNA] sequencing [of SARS-CoV-2] attributed to the place where they are actually doing a service to the world. (Canadian Researcher Respondent 21)

Equity considerations cut across COVID-19-related GHG and population health research priorities in Canada. Respondents further reported that equity within GHG and population health cannot be achieved if equity is not envisioned along with funding and accountability. According to them, the axis of funding, equity, and accountability is a crucial determinant for COVID-19-related GHG and population health.

You have to have dedicated resources to do that [global vaccine equity]. We [Canada] are one of the largest donors to Act A and we’ve made significant investments, for example in WHO in their health systems connector. We’ve made sure when we are negotiating these agreements we say OK, so here’s a budget line for you to do the equity analysis. (Canadian Research Donor Respondent 9)

Canada has contributed quite substantially to COVAX, at least rhetorically, on paper, we have made huge contributions and are committed to them. We haven’t delivered on promises and again, the public appetite for accountability is absent (…). There is no accountability or follow-up (…). And so, why does it happen that we have all of this ineffective governance? (Canadian Researcher Respondent 14)

Among papers included in the scoping review [13], equity considerations were both implicitly (Republic of Ireland, UK, India, South Africa, Australia, and the EU) [17, 19–22] and explicitly addressed related to socioeconomic (Brazil, France, USA, and Canada) [18, 23, 24], financial (BRICS) [25], gender (China, Hong Kong, UK, and Canada) [26], and health (BRICS, Japan, Italy, Singapore, China, Canada, and the EU) [25, 27–29] inequities, and climate resilience in Japan [30]. Furthermore, the papers that addressed explicitly equity considerations mostly discussed upstream determinants of health to respond to inequities, such as by implementing policies that focus on prevention [23] and economic factors and climate change [30], and addressing racism and sexism [26] and poor living conditions that increase the risks of contracting COVID-19 [18, 27].

### COVID-19-Related Consequences

The consequences of the COVID-19 pandemic are unprecedented and affected nations, population groups, service providers, policymakers, and researchers alike, worldwide. According to respondents, COVID-19 consequences were negative, positive, and unintended, and lessons were learned. One of the negative consequences addressed the intersectional inequities experienced by population groups that were already in situations of vulnerability.

We did a few research projects looking at the impact of COVID-19 on children with disabilities. The loss of access to services, to support. Families who depend on, for example, caregivers at home could not have access to the services. And those were essential for maintaining the family to work. (Canadian Researcher Respondent 19)

However, COVID-19-related consequences also brought positive aspects for researchers to collaborate and work differently.

It [COVID-19] has forced and enabled much more engagement between social science perspectives, epidemiological and clinical perspectives, public health perspectives, and mathematical modelling, and so I think that’s really interesting and important. It has highlighted platforms, information flows, and communications in ways that could have long-term positive consequences. (Canadian Researcher Respondent 35)

Consequences were not only negative and positive, but they were also unintended, for example, due to the explosion of research on COVID-19 being conducted, the role of social media as a channel for misinformation, and the exacerbation of inequities.

One [example] is just around the sheer pace of publication and the volume of publication. Preprints have absolutely exploded during COVID-19, and we have seen that when preprints are also accompanied by widespread press and social media attention that there can be some unintended consequences before the papers have gone through thorough peer review. I would say that issues of equity, diversity and inclusion within the research system have been exacerbated during the pandemic. (Canadian Research Donor Respondent 18)

Also, lessons have been learned about the need to consider more systematically equity as stated by one of our respondents: “The top three lessons of the pandemic I like to say is equity, equity, and equity.”, and through the importance of addressing COVID-19 through the One Health approach that promotes the interconnectedness of human, animal and environmental health and pandemic preparedness approaches.

Before the [COVID-19] pandemic, there were several international groups working on pandemic preparedness (…). Just in the past hundred years, we have got four influenza pandemics and we have two pandemics caused by coronaviruses (…), and of course to look at something that we call the One Health approach when we are thinking about the pandemic threat because over 75% of these emerging infectious diseases are coming from an animal source. (Canadian Research Centre Respondent 2)

The most common COVID-19 public health measures discussed in the papers included in the scoping review [13] were social and fiscal measures [17, 21, 25–27, 29, 30] to non-pharmaceutical interventions (NPI) that include stay-at-home instructions, physical distancing [17–21, 24, 25, 27, 28], and COVID-19 testing [19, 24–26]. Control measures such as lockdowns [20, 22, 23, 25–27] and pharmaceutical interventions (e.g., vaccines) [20, 25, 29] were also addressed. These measures affected most populations living and working in conditions of vulnerability and marginalisation. Studies conducted in Canada, the UK, and the EU reported that precarious workers, women, older adults, youth, racialized people, homeless people, people with disabilities, and LGBTQ+ communities were experiencing the brunt of COVID-19 public health measures because of their already vulnerable situations [19]. Domestic migrant workers in India further experienced economic and social discrimination, hunger, and death in the sudden aftermath of the national executive decision to implement a country-wide lockdown [20]. Women survivors of domestic violence in South Africa faced additional pressure to survive both violence and COVID-19 [21].

## Policy Options and Recommendations

In this section, we address the policy options and programmatic and research-related recommendations informed by evidence (Table 1). It is argued that policy options and recommendations for Canada and selected G20 countries are not mutually exclusive and are complementary to further improve COVID-19-related GHG and population and public health priorities, both at national and global levels.

**TABLE 1:** COVID-19-related GHG and population health policy options and programmatic and research recommendations in Canada and G20 countries^a^.

	Canada	Selected G20 countries including Canada
Policy options	• Provide sufficient funding for equitable and accountable global and public health outcomes	• Implement upstream determinants of health such as preventative policies that reduce inequities downstream
	• Train reviewers working for research funding agencies on equity	• More multilevel and intersectoral coordination and collaboration
	• Review the Act governing CIHR to address explicitly equity considerations	• Implement intersectional gender-focused policies in COVID-19 responses
Programmatic recommendations	• Public health schools/universities to train future decision-makers, researchers, and practitioners on equity (understanding, methods, metrics, and interventions) systematically	• Implement community needs assessments
	• Ensure data accessibility and operability enables equity-informed analysis, in real time	• Involve the community in COVID-19 response strategies development
		• Tailor interventions to local/country needs
Research recommendations	• Promote research that focuses on equity including interventional research	
	• Ensure research mobilisation and exchange foster policy change	

^a^ Note: The policy options and recommendations are not mutually exclusive but are rather complementary.

First, a key policy option reported by the Canadian-related qualitative study respondents as an important upstream determinant of both COVID-19 GHG and population health research is sufficient funding for equitable and accountable health outcomes to emerge meaningfully downstream, coupled with training of research grant reviewers on how equitable health outcomes look like for transformative change to happen subsequently. This policy option also concerns WHO whose mandate for global population health and health equity cannot be attained without adequate investment from the Member States. Another recommended key policy option by respondents was to review the Act that governs the Canadian Institutes of Health Research, an important health research funding agency in Canada, to address explicitly equity considerations in its Act beyond population health improvements as it is not currently the case [31]. This policy option is crucial as it has the great potential to stir the direction of population and public health research priorities in Canada through the equity lens. At the G20 level [13], policy options addressed the necessity for more multilevel and intersectoral coordination and collaboration [25, 29]. They also focused on upstream determinants of health that shape public health systems and governance structures, for example, by putting in place such as prevention-focused policies that reduce health inequities among vulnerable populations in France [23], an intersectional feminist perspective in COVID-19 responses in China, Hong Kong, Canada, and the UK [26], and gender equity-focused policies to avoid gender blind spots in pandemic responses in South Africa [21].

Second, to address equity considerations early among future leaders in population and public health or those potentially taking important population and public health and GHG decisions, Canadian-based study respondents suggested a specific programmatic recommendation for universities and schools of public health to include systematically equity considerations throughout their training curricula when training future global and/or public health practitioners, decision-makers, and researchers to prioritise equity throughout their training curricula. Another programmatic recommendation at the intersectoral level reported was the necessity to improve data accessibility and interoperability within and across sectors, for researchers and decision-makers to be able to analyse data using a systematic equity lens, in real time. Otherwise, ongoing equity-based research analyses would be missing. Programmatic recommendations at the G20 level [13] reported included interventions such as community needs assessment and evaluation of COVID-19-related service delivery in the EU [19] and the participation of population groups living and working in conditions of vulnerability and marginalisation in emergency response development in Brazil [18]. It was further suggested that prioritising the needs of communities and countries is essential to devise tailored COVID-19 response interventions in Italy, China, Singapore, and Japan [27]. Although the above recommendations are not completely new, they constitute a real opportunity for bringing about change, both at the Canadian and G20 countries’ levels.

Third, recommendations on research echoed policy options by both addressing upstream determinants of health and centering equity within research endeavours including intervention research, hence going beyond characterising problems and inequity issues (an area where most researchers excel in) in population health research. They specifically came from the Canadian-based qualitative study respondents. There is a call to address more complex global public health issues such as governance and equity, knowledge mobilisation and exchange including knowledge synthesis that need to explicitly contribute to health equity, especially for populations in conditions of vulnerability and marginalisation.

### Limitations

Perspectives of qualitative study respondents were collected between February and August 2022, hence they might have changed over time given the changed epidemiology of COVID-19 and the specific contexts of study respondents related to their geographic locations and public health measures put in place. With respect to the scoping review, only 14 studies met the inclusion criteria and were included in the last stage of the review. Our multiple inclusion criteria excluded studies that only examined one or partial concepts. It is also hypothesised that governance as an object of empirical study is still emerging and might have limited the inclusion of additional studies.

## Conclusion

This policy brief aimed to contribute to the evidence base on COVID-19-related GHG and population health research priorities in both Canada and selected G20 countries. Our findings corroborate lessons on COVID-19 reported in The Lancet Commission [1]. Global health governance and national governments failed to effectively address equity in COVID-19 policy, research, and programmatic responses. In spite of the negative consequences of the COVID-19 pandemic, programmatic and research opportunities exist along with concrete policy options that can be implemented without further delay. Upstream – structural – determinants of both global and national public health depend on “walking the talk,” systematically, for global health equity to become reality. When global health meets with local public health, global health equity is no longer a choice [32]. We collectively as decision- and policy-makers, researchers, representatives of civil society, and citizens share the duty for global health justice.
