# Supplementary material for: The Intersections of COVID-19 Global Health Governance and Population Health Priorities: Equity-Related Lessons Learned From Canada and Selected G20 Countries
Source: Public Health Rev. 2024 Jan 29;45:1606052. doi: 10.3389/phrs.2024.1606052 (PMC10859516; doi:10.3389/phrs.2024.1606052)
Supplement: Supplementary file 1 [file DataSheet1.pdf]

Annex 1. Search terms

Search Components	Search Keywords
COVID-19	COVID-19 or SARS-CoV-2 or Coronavirus Infections or coronavir or corona virus or betacoronavirs or covid19 or covid 19 or ncov or CoV 2 or cov2 or sarscov2 or 2019ncov or sars cov2 or sars-cov-2 or 2019 ncov or novel CoV
	AND
Global health governance	Global Health or government or internationality or international cooperation or global governance or government or collaboration or response or coordination or governance or government or policy or government or internationality or international cooperation or cooperation or partnership or coordination or leadership or global actors or state actors or non-state actors or global partnerships or global coordination or international obligations or national obligations or international assistance or international cooperation or intergovernmental or multilateral organizations or non-government organizations or institutions or governance arrangement
	AND
Population health	Health Promotion or Population Health or Public Health or population health or public health or epidemiology or preventive medicine or health promotion
	AND
Equity	Health Equity or Gender Equity or Social Determinants of Health or health status disparities or Social Justice or equit* or inequit* or equalit* or inequalit* or disparit* or social determinant of health or social health determinant* or SDH or social justice
	AND
G20 countries	G20 or Argentina or Australia or Brazil or Canada or China or France or Germany or India or Indonesia or Italy or Japan or Mexico or Russia or Saudi Arabia or South Africa or South Korea or Turkey or UK or United Kingdom or US or USA or United States of America or EU or European Union

Source: Mac-Seing M, Gidey M, Di Ruggiero E. COVID-19-related global health governance and population health priorities for health equity in G20 countries: a scoping review. International Journal for Equity in Health. 2023;22(1):232.

Annex 2. Flow chart

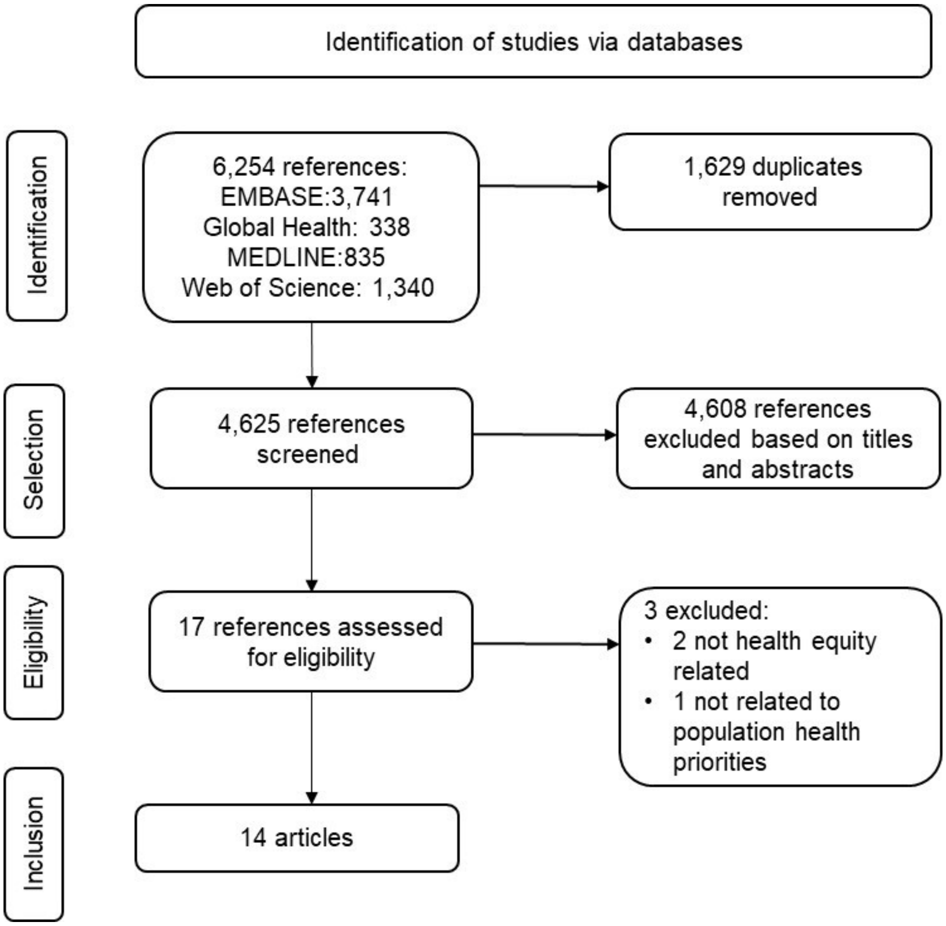

Source: Mac-Seing M, Gidey M, Di Ruggiero E. COVID-19-related global health governance and population health priorities for health equity in G20 countries: a scoping review. International Journal for Equity in Health. 2023;22(1):232.
